# A Tooth in the Tongue

**Published:** 1875-07

**Authors:** 


*A Tooth in the Tongue.*—The following is being circulated by the newspapers:

The Houston, Ga. *Home Journal* says Mr. Wm. Brunson had a tooth an inch long extracted from the centre of his tongue near the root, one day last week. It was imbedded in the muscles, and entirely disconnected from the jaws or gums.

The two articles published in June number of JOURNAL, from the pens of Drs. Cushing and Turner, on "What our patients may Expect," and "Plaster Impressions," were taken from Volume of Proceedings of N. Y. Odontological Society, and so credited, which was prepared and published by S. S. White for *Cosmos.*

---

### OBITUARY.

